# The relationship between social support and psychological crisis vulnerability among family impoverished undergraduates: the intermediary role of psychological resilience

**DOI:** 10.3389/fpubh.2025.1501513

**Published:** 2025-01-27

**Authors:** Weimin Yuan, Jinhui Ning, Mengke Huo, Yiwei Feng

**Affiliations:** College of Economics and Management, Hebei Agricultural University, Baoding, China

**Keywords:** family impoverished undergraduates, mental health, social support, psychological crisis vulnerability, psychological resilience, intermediary role

## Abstract

**Background:**

Family impoverished undergraduates are more likely to be vulnerable to psychological crises. Therefore, this study aimed to elucidate the intrinsic relationship between social support, psychological resilience, and psychological crisis vulnerability among family impoverished undergraduates and to provide valuable practical evidence and policy support for effectively improving the mental health of family impoverished undergraduates.

**Methods:**

This study applied quantitative methods to explore the impact of social support on psychological crisis vulnerability among family impoverished undergraduates while also examining the mediating effect of psychological resilience in this process. First, we used the Psychological Crisis Vulnerability Measurement Questionnaire to assess the psychological crisis vulnerability among family impoverished undergraduates and compared it under different demographic characteristics. Second, we expanded the independent variables that affect psychological crisis vulnerability among family impoverished undergraduates and conducted multiple linear regression analyses. Finally, we employed a structural equation modeling approach to analyze the underlying mechanisms between social support and psychological crisis vulnerability and to test the conspicuousness of the mediating role of psychological resilience in the process by which social support affects psychological crisis vulnerability.

**Results:**

Analysis of 1,549 valid questionnaires revealed that the overall level of psychological crisis vulnerability among family impoverished undergraduates was moderate. Among the surveyed family impoverished undergraduates, 37.62% reported difficulty in maintaining psychological balance when facing crises, and 4.73% showed a very vulnerable response to crises. Factors significantly associated with the psychological crisis vulnerability of family impoverished undergraduates included being a student from a financially disadvantaged background, coming from a single-parent family, and having experienced being a left-behind child. Additionally, the participants had a social support score of 60.29 ± 11.31, indicating that individual social support was significantly correlated with family support, friend support, and support from significant others. This study confirms that social support can indirectly predict psychological crisis vulnerability of family impoverished undergraduates through psychological resilience.

**Conclusion:**

Family impoverished undergraduates are more prone to psychological crises, and effective social support can help transform them into motivational forces for their own development. This, in turn, can improve their psychological state when facing problems, and enhance their ability to solve them. Furthermore, social support influences the psychological crisis vulnerability of family impoverished undergraduates through psychological resilience. Therefore, we suggest that universities pay attention to the developmental needs of students’ subjectivity, strengthen their psychological and behavioral training, further improve psychological counseling stations, and provide group counseling to students, as appropriate. Simultaneously, attention should also be paid to students’ family issues, helping create a positive family environment for students and strengthening home-university cooperation to effectively intervene in undergraduates’ psychological crises.

## 1 Introduction

With the continuous progress of society and the ongoing optimization of government funding systems, the extreme poverty faced by undergraduates worldwide have significantly improved. However, because of regional disparities and uneven economic development among families, economic difficulties faced by undergraduates still exist in various ways (1). The comprehensive and healthy development of family impoverished undergraduates not only helps improve the livelihood of underprivileged families but also effectively prevents the intergenerational transmission of poverty, while positively affecting social equity and progress. Therefore, the governments of countries place high importance on this vulnerable group with significant potential for development and are committed to ensuring their physical and mental health and comprehensive development (2). In China, the proportion of impoverished undergraduates accounts for nearly 20% of the total; in some regions, this proportion even reaches 50%. Owing to limitations in their living environment and economic conditions, especially when they are facing multiple pressures such as social interactions, academic workload, and career competition, their psychological susceptibility and crisis tendencies may become more apparent (3, 4). A 2023 statistical report indicates that the proportion of undergraduates in China with mild emotional distress exceeds 10%. Among undergraduates with severe depression, the incidence is 3.17% in men and 5% in women (5).

Psychological crises, a natural response to stress, typically occur when individuals encounter unexpected events or significant issues. When an individual’s usual coping mechanisms and support networks fail to function effectively, it can lead to psychological imbalance. Psychological vulnerability encompasses susceptibility to adversity, the capacity to cope with it, and the resilience to recover from it. Individuals with higher psychological vulnerability are more likely to experience psychological imbalances when faced with stress. Given the significant role that psychological crisis vulnerability plays in predicting potential mental health issues, it has become a popular topic in mental health research (6). Simultaneously, as a regulatory mechanism for promoting mental health, social support is gradually playing its role in higher education institutions and has become an important component of undergraduates’ mental health development (7). Social support can be understood as a social system comprising individuals and the social groups with which they have social relationships as well as the social connections between these individuals and groups. Malecki et al. (8) believe that the strength of an individual’s social support is significantly related to the degree of family poverty, that social support can effectively alleviate adverse emotional responses caused by stress, and that good social support is negatively correlated with psychological problems among college students. For instance, through an exploration of the relationship between different levels of social support among undergraduates and melancholia, Pratyusha et al. (9) suggest that the social support received by college students can effectively reduce the likelihood of melancholia. This further indicates that good social support can have effects similar to formal psychotherapy.

Psychological resilience is a key element that enables individuals to fully utilize their personality traits when dealing with crises, enhance their problem-solving abilities, and effectively cope with and resolve crises (10). As defined by the American Psychological Society, psychological resilience reflects an individual’s capacity to recover and bounce back when encountering stress and setbacks. Therefore, individuals with higher levels of psychological resilience will be able to effectively cope with life’s stresses and challenges, thereby maintaining or improving their psychological well-being. In turn, there is a strong correlation between an individual’s psychological resilience and the social support he or she receives. Research has shown that there is a significant positive correlation between social support and psychological resilience, i.e., the level of social support has a direct impact on an individual’s ability to adapt in the face of adversity (11). This implies that social support can facilitate individuals build positive coping strategies and bolster their problem-solving skills, and as a result, fortify their psychological resilience.

In summary, social support not only directly positively affects individuals’ mental health, but also bolsters their psychological resilience by providing emotional and informational support, thus indirectly improving their ability to cope with adversity and ultimately alleviating and reducing their psychological crisis vulnerability. Consequently, it is imperative to introduce the element of psychological resilience into the study of the relationship between social support and the psychological crisis vulnerability among family impoverished undergraduates. However, research on the interrelationships between social support, psychological resilience, and psychological vulnerability is notably scarce among family impoverished undergraduates. Therefore, this study hypothesized that social support can positively affect the psychological crisis vulnerability of family impoverished undergraduates through the mediating role of psychological resilience. Based on this, the main goal of this study is to attempted to elucidate the internal influence mechanisms between social support and psychological crisis vulnerability among family impoverished undergraduates. Furthermore, it explored the policy measures that can enhance the psychological well-being of family impoverished undergraduates and provide valuable theoretical and practical support for mental health education for this group.

## 2 Methods

### 2.1 Study design

This study quantitatively analyzed the role of social support in psychological crisis vulnerability among family impoverished undergraduates and how psychological resilience mediates this relationship. First, we used the Psychological Crisis Vulnerability Measurement Questionnaire to assess the psychological crisis vulnerability among family impoverished undergraduates and employed Harman’s single-factor test method to evaluate the common source variance in the data. Second, we compared psychological crisis vulnerability across demographic characteristics. Based on the comparison results, we used the scores from the Psychological Crisis Vulnerability Measurement Questionnaire as the dependent variable. The independent variables in this study were further expanded to include family structure, impoverished undergraduates,^1^ fathers’ educational level, experience of being a left-behind child, trust and encouragement, emotional warmth, indulgence, and neglect (12). Subsequently, a multiple linear regression analysis was conducted on these variables. Finally, based on the research hypotheses, a structural equation model was employed to construct path relationships between the variables to test the mediating effect of psychological resilience. Furthermore, this study utilized the Bootstrap method to extract sample data, and a significance analysis was conducted to explore the mediating effect of psychological resilience as a moderating variable between social support and psychological crisis vulnerability of family impoverished undergraduates at the 95% confidence level.

### 2.2 Recruitment and sampling

This study, conducted from July to August 2024, used cluster sampling to select four universities in Baoding City, Hebei Province, as the research sample areas. Three colleges were selected randomly from each university. Subsequently, a sampling questionnaire survey was conducted among full-time undergraduate students from all grades at these colleges. The questionnaire instructions clearly stated the purpose of the survey, emphasized the anonymity of the responses, and ensured that participants had a comprehensive understanding of the study. During the course of the study, the total number of students surveyed exceeded 1,600, and a total of 1,600 questionnaires were distributed. After excluding questionnaires that did not meet the filling requirements, 1,549 valid questionnaires were collected (the main characteristics of the respondents are shown in the Table 1), with a recovery rate of 96.8%. All participants voluntarily participated in the questionnaire survey.

**Table 1:** Comparison of undergraduates’ psychological crisis vulnerability with different demographic characteristics.

Demographic indicators	Options	Number of people	Psychological crisis vulnerability	*t/F*-value	*p*-value
Gender	Man	809	10.77 ± 3.04	0.41	0.71
	Woman	740	10.75 ± 2.93		
Major type	Humanities	645	10.82 ± 3.26	1.03	0.32
	STEM	904	10.72 ± 3.13		
University year	Freshman year	508	10.78 ± 3.16	0.22	0.86
	Sophomore year	390	10.78 ± 2.84		
	Junior year	403	10.73 ± 3.11		
	Senior year	248	10.72 ± 3.42		
Family structure	Two-parent family	1,359	10.70 ± 3.31	3.25	<0.01
	Single-parent family or other	190	11.13 ± 2.71		
Only child	Yes	654	10.81 ± 3.14	0.91	0.36
	No	895	10.71 ± 3.21		
Impoverished undergraduates	Yes	268	11.28 ± 2.56	4.39	<0.01
	No	1,281	10.64 ± 3.39		
Father’s education level	Junior high school and below education	735	10.91 ± 3.13^﹡^	3.91	0.02
	Vocational secondary or high school diploma	584	10.82 ± 3.07^﹡^		
	Higher vocational education or above	230	10.39 ± 2.73		
Mother’s education level	Junior high school and below education	875	10.90 ± 3.40	0.51	0.60
	Vocational secondary or high school diploma	528	10.77 ± 3.15		
	Higher vocational education and above	146	10.61 ± 2.60		
Left-behind child	Yes	491	11.12 ± 3.10	5.85	<0.01
	No	1,058	10.50 ± 3.12		

Comparison of the educational levels of fathers with higher vocational education and above, *p* < 0.05.

### 2.3 Data collection

A semi-structured interview guide developed by the research team was used. The primary method of data collection was one-on-one, face-to-face questionnaire surveys, complemented by the distribution through the “Questionnaire Star” platform (with 37.89% of the questionnaires being distributed electronically). The following data collection tools were used.

#### 2.3.1 Basic information survey questionnaire

The basic information survey questionnaire in this study covered the participants’ demographic information, including but not limited to age and gender; type of family location (urban or rural); educational level of parents; field of expertise studied (e.g., economic management, engineering technology, medicine, or arts); current academic year; whether they were identified as impoverished by the college/university; whether they had received financial assistance from the college/university; family structure (single-parent family or other); and personal health status (whether they had ever used psychotropic medication).

#### 2.3.2 Impoverished family status questionnaire

This study drew lessons from the impoverished family standards set by the China Education Tracking Survey (2013–2014) and refers to the findings of scholars such as He Guangye. It identified students’ family economic status by asking questions such as “How would you evaluate your family’s financial situation?” to determine whether they fell within the category of family poverty (13). Specifically, this study used students’ self-assessments of their families’ economic situations as the basis for determining their poverty status. If a student rated their family’s economic situation as “poor” or “very poor,” their family was classified as being impoverished. Conversely, if the evaluation was “average,” “good” or “excellent,” the family was considered not to be impoverished. Owing to the potential for error in students’ self-assessments, this study considered the following key factors when assessing the poverty status of students’ families: On the one hand, there are significant differences in economic development and consumption levels between different regions and between urban and rural areas in China; on the other hand, family income is often difficult to measure accurately, and students’ understanding of their families’ economic situations is likely to be partial and limited. Therefore, the judgment in this study can only be established based on the subjective evaluations of students from different regions.

#### 2.3.3 Psychological crisis vulnerability scale

This study referred to the Vulnerability Assessment Scale revised by Wenmin Li et al. (14). The scale covers 15 assessment items, each with three response options: “Definitely,” “Possibly,” and “Definitely Not,” and each is assigned a score ranging from 0 to 2 points. As per the scale, the degree to which an individual is affected by psychological crises can be divided into four levels: a score below 5 points indicates that the individual has very high resistance to crises and is almost unaffected by them, a score between 5 and 10 points indicates that the individual has strong coping abilities and can successfully handle most crises, a score between 11 and 15 points implies that the individual may face challenges in maintaining psychological balance in certain crises, and a score above 15 points suggests that the individual is extremely vulnerable in the face of crises and has difficulty maintaining psychological stability. In previous studies, scholars such as Zengrang Luo applied the Crisis Vulnerability Test Scale to assess the psychological crisis vulnerability of domestic master’s degree candidates. Luo’s research results indicated that the scale possessed a high degree of internal consistency, with a Cronbach’s alpha coefficient of 0.71.

#### 2.3.4 Social support scale

We used the Social Support Scale developed by Zimet et al. and revised by Peiwan Fang et al. to measure the level of social support among college students (15). The scale consists of 12 items, with each item offering five response options ranging from “completely disagree” to “completely agree,” with the corresponding scores ranging from 1 to 5 points. The Social Support Scale used in this study covers three dimensions—family, friends, and key figures^2^—to assess the degree of social support perceived by an individual. The scale score reflects an individual’s ability to identify and utilize external support resources, with higher scores indicating greater capabilities. In this study, the scale demonstrated good internal consistency with a Cronbach’s α coefficient of 0.925. The internal consistency of the subscales was also good, with the family support dimension having a Cronbach’s α coefficient of 0.839, friend support dimension having a Cronbach’s α coefficient of 0.849, and key figures support dimension having a Cronbach’s α coefficient of 0.844.

#### 2.3.5 Psychological resilience scale

We utilized the Psychological Resilience Scale developed by Connor, refined by Campbell-Sills et al. (CD-RISC), and translated into Chinese by Zengjie Ye (16). All items of the scale use a 5-point Likert scoring system, with ratings ranging from “completely disagree” to “completely agree,” and scores ranging from 1 to 5. The higher the score, the greater the individual’s ability to self-regulate in the face of adversity. In this study, the scale demonstrated a high level of internal consistency, with the Cronbach’s α coefficient reaching 0.887.

## 3 Results

The data entry in this study was completed using Excel spreadsheets and underwent dual verification by two researchers to ensure accuracy. A series of statistical analyses were conducted on the research data using SPSS 26.0 software, including ANOVA, *t*-tests, correlation analysis, and multiple linear regression. Based on the research hypothesis, a structural equation model was constructed using AMOS 24.0 software. At a 95% confidence level, the significance of the mediating effect of psychological resilience between social support and psychological crisis vulnerability in family impoverished undergraduates was tested using the Bootstrap method (with 2000 resamples).

### 3.1 Psychological crisis vulnerability scores

The average score for psychological crisis vulnerability among family impoverished undergraduates was 10.76 (with a standard deviation of 3.46). Further analysis revealed that 5% of undergraduates (77 individuals) scored below 5, 47.25% (732 individuals) scored between 5 and 10, 41.85% (648 individuals) scored between 11 and 15, and 5.90% (92 individuals) scored above 15.

### 3.2 Common method bias test

This study employed Harman’s single-factor test to assess the potential common method bias. By conducting an unrotated exploratory factor analysis, we identified four factors with eigenvalues greater than 1. The degree of interpretation of the primary factor of variability was 28.13%, which is below the critical threshold of 40%. This finding suggests that there was no significant common method bias in the data.

### 3.3 Comparison of psychological crisis vulnerability across demographic characteristics

According to the analysis results, impoverished undergraduates from single-parent families or other non-traditional family structures exhibited higher scores for psychological crisis vulnerability than those from two-parent families. Additionally, impoverished undergraduates scored higher on psychological crisis vulnerability than their non-impoverished counterparts. In terms of educational background, undergraduates whose fathers had a diploma in higher vocational education or above exhibited significantly lower psychological vulnerability scores than those whose fathers had lower levels of education. Undergraduates who had been left-behind children scored significantly higher than those without such experiences (*p* < 0.05). However, factors such as gender, grade level, field of study, only child status, and mother’s education level did not show statistically significant differences in the psychological crisis vulnerability scores of family impoverished undergraduates (*p* > 0.05) (Table 1).

### 3.4 Multiple linear regression analysis

Based on the analysis results presented in Table 1, this study retained the indicators with *p*-values <0.05. Additionally, using the scores from the psychological crisis vulnerability assessment questionnaire as the dependent variable and integrating the findings from the study by Hu Fan et al. (12), we expanded the variables influencing the psychological crisis vulnerability of family impoverished undergraduates to the following nine: family structure, impoverished undergraduates, father’s educational level, experience of being a left-behind child, trust and encouragement, emotional warmth, pampering, and neglect. These variables were then included in the multiple linear regression analysis. The results indicated that among the undergraduates, those from single-parent families, identified as impoverished students, who had experienced being a left-behind child, or who were excessively pampered or neglected during their upbringing, tended to score higher on psychological crisis vulnerability. This suggests that these individuals may be more vulnerable to stress. Conversely, when these students experienced higher levels of trust and encouragement, as well as greater emotional support and warmth, their psychological vulnerability scores tended to be relatively low (with *p*-values <0.05). This may have contributed to their ability to maintain greater psychological resilience in the face of adversity (Table 2).

**Table 2:** Results of multiple linear regression analysis (*n*=1,549).

Independent variable	Options	Standard error	*T-*value	*p-*value	*β*-value (95% CI)
Family structure	Single-parent family or other	0.09	4.16	<0.01	0.28 (0.12–0.33)
Impoverished undergraduates	Yes	0.08	2.50	0.01	0.25 (0.17–0.49)
Father’s education level	Junior high school and below education	0.60	1.82	0.07	0.91 (−0.22–2.10)
	Vocational secondary or high school diploma	0.38	1.55	0.11	0.62 (−0.17–1.35)
Left-behind child	Yes	0.08	7.50	<0.01	0.38 (0.12–0.67)
Trust and encouragement		0.03	−7.32	<0.01	−0.14 (−0.74–-0.09)
Emotional warmth		0.03	−8.50	<0.01	−0.19 (−0.50–-0.07)
Pampering		0.03	6.66	<0.01	0.23 (0.17–0.56)
Neglect		0.03	5.78	<0.01	0.21 (0.09–0.83)

The family structure variable is referenced with two-parent families; impoverished undergraduates are referenced with non-impoverished undergraduates; father’s educational level is referenced with those who have higher vocational education or above; experiences of being a left-behind child are referenced with those who were not; trust and encouragement, emotional warmth, pampering, and neglect are treated as continuous variables. The coefficient of determination *R*^2^ = 0.65.

### 3.5 Structural equation modeling analysis

**Figure 1:**
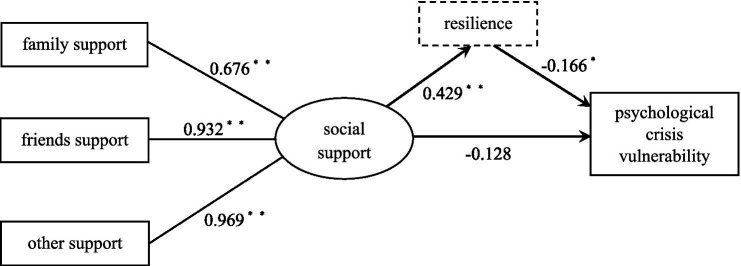
Relationship model diagram of social support, psychological resilience, and vulnerability to psychological crises. *p<0.05; **p<0.01; ***p<0.001.

To further explore how social support affects vulnerability to psychological crises and to validate the mediating role of psychological resilience, this study used structural equation modeling based on the research hypothesis to depict the interrelationships and pathways of action among various variables (Figure 1). The fitting index results for the model are as follows: *x*^2^/*df* = 1.798, GFI = 0.919, AGFI = 0.929, IFI = 0.979, CFI = 0.971, TLI = 0.969, RMSEA = 0.035, and SRMR=0.037. The results of the data analysis confirmed a good fit between the model and sample data. Further analysis of the paths in the structural equation model revealed that the three dimensions of social support (family support, friend support, and significant others’ support) explained 67.6, 93.2, and 96.9% of social support, respectively, with all *p*-values<0.001, thereby demonstrating the effectiveness of these three dimensions in explaining social support. Specifically, social support significantly and positively predicted psychological resilience (*β* = 0.429, *p* < 0.001) and significantly and negatively predicted vulnerability to psychological crises (*β* = − 0.128, *p* = 0.028), whereas psychological resilience significantly and negatively predicted vulnerability to psychological crises (*β* = −0.166, *p* = 0.002).

### 3.6 Mediation effect analysis

In this study, we adopted the Bootstrap method to repeatedly sample the data 2000 times, using a 95% confidence level as the basis, to conduct significance testing for psychological resilience as a mediating variable in the influence of social support on the psychological crisis vulnerability of family impoverished undergraduates. The confidence intervals obtained through the bias-corrected and percentile methods excluded the value of 0, demonstrating the statistical significance of the mediating effect (Table 3). In addition, the impact of social support on psychological crisis vulnerability was significantly negatively correlated, with the effect value of −0.108 and the corresponding significance level of *p* = 0.019. Moreover, psychological resilience as a mediating variable was also confirmed to have a significant effect, with the effect value of −0.062 and the significance level of *p* = 0.003.

**Table 3:** Path and effect testing of social support on psychological crisis vulnerability.

Path relationships	Effect value	Standard error	Bias-corrected 95% confidence interval		Percentile 95% confidence interval		*p*-value
			Lower limit	Upper limit	Lower limit	Upper limit	
Social support — psychological crisis vulnerability	−0.108	0.040	−0.133	−0.068	−0.128	−0.072	0.019
Social support — psychological resilience — psychological crisis vulnerability	−0.062	0.025	−0.068	−0.015	−0.068	−0.009	0.003

## 4 Discussion

### 4.1 Insights derived from the model analysis results

#### 4.1.1 Overall level of psychological crisis vulnerability among family impoverished undergraduates

The results of the survey scale in this study indicate that impoverished undergraduates exhibit a moderate level of psychological crisis vulnerability. Specifically, 37.62% of these students often found it difficult to maintain psychological stability when facing crises, and 4.73% appeared extremely vulnerable in the face of such crises. This study revealed that family impoverished undergraduates exhibited a higher level of susceptibility and vulnerability to psychological crises. In this regard, the results of comparison of psychological crisis vulnerability across demographic characteristics indicate that the psychological crisis vulnerability of students from single-parent families, impoverished undergraduates, fathers with lower levels of education, and those with experience of being left behind exhibit more pronounced psychological crises vulnerability. First, impoverished undergraduates from single-parent families are affected by changes in family structure and family dysfunction, resulting in a relative lack of family supervision and education for their children, which may lead to psychological problems such as sensitivity, anxiety and low self-esteem; Second, the economic situation of impoverished college students to some extent restricts their social interactions and consumption abilities, forcing them to carefully consider expenditures for socializing and gatherings, and even worrying about being ridiculed by classmates for their economic capabilities, which in turn leads to their sensitivity to interpersonal interactions, and thus to potentially causing social withdrawal and self-isolation. In addition, the predictable pressure of employment may also lead to further anxiety and depression (17, 18). Third, the lower father education levels may result in the absence of the father’s role and inadequate emotional support, which may cause children to have corresponding behavioral issues and affect their cognitive ability, potentially resulting in feelings of loneliness, anxiety, and low self-esteem; Fourth, the prolonged experiences of being left behind may cause children to feel lonely and neglected due to a lack of parental companionship, which in turn to affect their social adaptability, causing them to avoid or fear social activities, and even manifesting as paranoia, impulsiveness, and sensitivity in interpersonal relationships.

#### 4.1.2 Factors influencing psychological crisis vulnerability among family impoverished undergraduates

First, the results of multiple linear regression analysis indicate that undergraduates from single-parent families or other non-traditional family structures, impoverished undergraduates, and those who have experienced being a left-behind child show the higher level of psychological vulnerability. As mentioned earlier, this may be because undergraduates from single-parent families or other non-traditional family structures experience a lack of parental roles during their upbringing, which can lead to insufficient emotional communication with their parents, resulting in a lack of effective support from their family (19, 20). Impoverished undergraduates are often very sensitive to their families’ economic situations, which may lead to a pervasive sense of anxiety and helplessness stemming from a desperate desire to escape poverty. This can cause them to experience feelings of failure and inferiority in social interactions, subsequently adversely affecting their mental health (21, 22). Undergraduates who have experienced being left-behind children may have underdeveloped personalities during their childhood due to the lack of parental companionship and proper educational guidance (23). Consequently, they may lack appropriate ways to express themselves and effective communication skills when facing problems, leading to significant psychological pressure (24, 25).

Second, the social support score of the 1,549 undergraduates in this study was 60.29 ± 11.31 points. By conducting a path analysis on the three subdimensions of social support, we found that the social support received by individuals was highly positively correlated with family support, friend support, and support from key figures. Moreover, the hypothesis that social support directly predicts psychological crisis vulnerability was confirmed. Social support can be subtly transformed into a motivating force for individuals, and this positive effect helps them maintain an optimistic attitude toward adversity and enhances their ability to overcome difficulties (26, 27).

Finally, this study confirmed that there is an indirect pathway through which social support affects the psychological crisis vulnerability of family impoverished undergraduates, indicating that social support can indirectly predict this through psychological resilience. When individuals perceive support from their external environment, it can promote the development of their self-affirmation and inspire greater inner strength, thereby encouraging them to face challenges with a positive mindset and approach.

### 4.2 Strategies and recommendations based on the quantitative analysis results

Promoting the comprehensive development of students’ physical and mental health is a major issue of concern for governments worldwide. The incidence of psychological problems among undergraduates has significantly increased, and the age of onset is decreasing, resulting in a notable rise in extreme cases triggered by these issues (28). Based on this analysis, this study proposes the following strategies and recommendations.

First, it is important to prioritize the developmental needs of undergraduate students’ subjectivity and strengthen their psychological and behavioral training. Colleges and universities should pay attention to the personalized psychological developmental needs of undergraduates. They can provide students with a variety of content-rich mental health services, including self-identity, career development, social skills, and emotional management, through methods such as book clubs, psychological workshops, group counseling, individual consultations, and skill-enhancement training. Simultaneously, it is advisable to advocate for the use of group teaching and experiential learning methods to effectively integrate psychological and behavioral training into classroom instruction. This will help students master scientific psychological intervention techniques and develop stable positive psychological behavioral patterns.

Second, attention should be paid to family issues to create a positive family environment. Family relationships and the family environment serve as important emotional support for college students and directly impact their mental health (29). Parents must maintain good communication with their college-aged children and create a family environment filled with love and security to promote a focus on academic pursuits and personal growth. In addition, family members should actively communicate and participate in family activities together, creating a harmonious atmosphere of mutual respect and understanding that provides undergraduates with the necessary emotional support and promotes their mental health and overall development.

Finally, improving psychological counseling stations and providing appropriate group counseling is essential. Facing significant changes in their academic and living environments, family impoverished undergraduates may experience considerable psychological adaptation pressures (30). During this period, universities should identify the psychological needs of undergraduates, establish detailed individual psychological profiles, and foster positive interactive relationships in a timely manner to help them overcome their initial feelings of discomfort. Additionally, psychological counseling stations commonly established in Chinese universities should accurately identify the mental health issues of family impoverished undergraduates and, through the provision of customized psychological counseling, help them adapt to university life more quickly and effectively.

### 4.3 Conclusions, strengths and limitations of this study

The results of this study indicate that family impoverished undergraduates are more prone to psychological crises, and effective social support can help transform them into motivational forces for their own development. This, in turn, can improve their psychological state when facing problems, and enhance their ability to solve them. Furthermore, social support influences the psychological crisis vulnerability of family impoverished undergraduates through psychological resilience. In this regard, we suggest that universities should pay more attention to the developmental needs of students’ subjectivity, strengthen their psychological and behavioral training, further improve psychological counseling stations, and provide group counseling to students, as appropriate. Simultaneously, attention should also be paid to students’ family issues, helping create a positive family environment for students and strengthening home-university cooperation to effectively intervene in undergraduates’ psychological crises.

This study revealed the interaction mechanisms between social support, psychological crisis vulnerability, and psychological resilience among family impoverished undergraduates. It provides a scientific basis for governments and universities to implement more targeted mental health interventions and offers valuable empirical support and policy references for further improvements in mental health education. However, this study has certain limitations that warrant further research. First, as a cross-sectional study, the data collected during the research process are all temporal, and most come from the subjective evaluations of the respondents, which limits the ability to more deeply elucidate the relationship between undergraduates’ psychological crisis vulnerability and related influencing factors. Second, this study is limited to Baoding City and does not encompass a broader range of sample collection. Therefore, future research could consider expanding the scope of the study based on the circumstances of different regions and student populations, as well as incorporating a wider array of indicators in the evaluation process.

## Data Availability

The original contributions presented in the study are included in the article/Supplementary material, further inquiries can be directed to the corresponding author.

## Footnotes

^1^In this study, “impoverished undergraduates” refers to those students who are identified by their schools as coming from families in financial hardship and in need of economic assistance.

^2^In this study, the terms “family,” “friends,” and “key figures” primarily refer to family members, classmates, and teachers, respectively.
