# Supplementary material for: The relationship between social support and psychological crisis vulnerability among family impoverished undergraduates: the intermediary role of psychological resilience
Source: Front Public Health. 2025 Jan 27;13:1501513. doi: 10.3389/fpubh.2025.1501513 (PMC11807794; doi:10.3389/fpubh.2025.1501513)
Supplement: Supplementary file 1 [file Table_1.DOC]

**Supplemental material 1: Psychological Crisis Vulnerability Scale**

| **Test questions** | **Definitely** | **Possibly** | **Definitely Not** |
| --- | --- | --- | --- |
| 1.I don't mind if the daily order of study is interrupted by unexpected events. | 0 | 1 | 2 |
| 2.Life is full of interesting adventures. | 0 | 1 | 2 |
| 3.Every problem has its solution. | 0 | 1 | 2 |
| 4.By consciously planning daily life, potential crises can be effectively prevented. | 0 | 1 | 2 |
| 5.I firmly believe that I can achieve what I pursue by studying hard. | 0 | 1 | 2 |
| 6.Even in the face of difficulties and challenges, I still have the autonomy to make my own choices. | 0 | 1 | 2 |
| 7.I enjoy listening to others share their experiences and insights. | 0 | 1 | 2 |
| 8.I can't study diligently because it always seems like others benefit from my efforts. | 0 | 1 | 2 |
| 9.Once authorities reach a conclusion on a matter, I tend to believe that I cannot change it. | 0 | 1 | 2 |
| 10.Most of the difficulties I encounter stem from my own improper actions. | 0 | 1 | 2 |
| 11.The dissatisfaction of others will make me feel very frustrated and unhappy. | 0 | 1 | 2 |
| 12.When others make excessive requirements of me, I find it hard to change their minds. | 0 | 1 | 2 |
| 13.Those whom I trust and rely on often leave me feeling disappointed. | 0 | 1 | 2 |
| 14.As long as I avoid difficulties, those problems will cease to exist. | 0 | 1 | 2 |
| 15.I find that people generally do not appreciate everything I do for them. | 0 | 1 | 2 |

**Supplemental material 2: Social Support Scale**

| **Test questions** | **Completely disagree** | **Relatively disagree** | **Uncertain** | **Relatively agree** | **Completely agree** |
| --- | --- | --- | --- | --- | --- |
| 1.When I encounter difficulties, my family, classmates, or teachers often accompany me. | 1 | 2 | 3 | 4 | 5 |
| 2.I can enjoy happiness with my family, classmates, or teachers, and also undertake sadness with them. | 1 | 2 | 3 | 4 | 5 |
| 3.My family is always able to provide me with substantial support to help me solve various problems. | 1 | 2 | 3 | 4 | 5 |
| 4.When facing difficulties, my family is always able to provide me with the necessary emotional support. | 1 | 2 | 3 | 4 | 5 |
| 5.When I encounter difficulties, my family, classmates, and teachers often become my spiritual pillars. | 1 | 2 | 3 | 4 | 5 |
| 6.My friends are always able to provide me with practical help. | 1 | 2 | 3 | 4 | 5 |
| 7.My classmates are always trustworthy when I encounter difficulties. | 1 | 2 | 3 | 4 | 5 |
| 8.I am able to discuss the problems I encounter in depth with my family. | 1 | 2 | 3 | 4 | 5 |
| 9.My classmates are always willing to share the joys and sorrows of life with me. | 1 | 2 | 3 | 4 | 5 |
| 10.In my daily life, my family, classmates, and teachers all care about my emotions and feelings. | 1 | 2 | 3 | 4 | 5 |
| 11.When I need to make choices, my family always voluntarily helps me and supports my decisions. | 1 | 2 | 3 | 4 | 5 |
| 12.I am able to have in-depth discussions with my classmates about the challenges I encounter. | 1 | 2 | 3 | 4 | 5 |

**Supplemental material 3: Psychological Resilience Scale**

| **Test questions** | **Completely disagree** | **Relatively disagree** | **Uncertain** | **Relatively agree** | **Completely agree** |
| --- | --- | --- | --- | --- | --- |
| 1.Experiences of failure often lead to a blow to my confidence. | 1 | 2 | 3 | 4 | 5 |
| 2.I often find it difficult to manage my negative emotions. | 1 | 2 | 3 | 4 | 5 |
| 3.I usually become more mature and experienced after experiencing failure. | 1 | 2 | 3 | 4 | 5 |
| 4.I tend to doubt my abilities after experiencing failure. | 1 | 2 | 3 | 4 | 5 |
| 5.When encountering something unpleasant, it is difficult for me to find the right person to confide. | 1 | 2 | 3 | 4 | 5 |
| 6.I have peers of the same age who are willing to listen to my difficulties. | 1 | 2 | 3 | 4 | 5 |
| 7.My opinions and ideas are always respected by my parents. | 1 | 2 | 3 | 4 | 5 |
| 8.I believe that process is more conducive to personal growth than outcome. | 1 | 2 | 3 | 4 | 5 |
| 9.When encountering difficulties, I usually create a plan and formulate specific measures. | 1 | 2 | 3 | 4 | 5 |
| 10.I tend to hide my inner feelings rather than share them with others. | 1 | 2 | 3 | 4 | 5 |
| 11.I believe that challenges can inspire people's fighting spirit. | 1 | 2 | 3 | 4 | 5 |
| 12.My thoughts are often intervened by my parents. | 1 | 2 | 3 | 4 | 5 |
| 13.At home, my opinions and viewpoints are often overlooked. | 1 | 2 | 3 | 4 | 5 |
| 14.My parents do not provide me with sufficient confidence and emotional support. | 1 | 2 | 3 | 4 | 5 |
| 15.When facing difficulties, I will take the initiative to seek help from others. | 1 | 2 | 3 | 4 | 5 |
| 16.I can always focus on overcoming difficulties when I meet challenges. | 1 | 2 | 3 | 4 | 5 |
| 17.I usually need a long time to let go of unpleasant experiences. | 1 | 2 | 3 | 4 | 5 |
| 18.My parents often motivate me to do my best to achieve my goals. | 1 | 2 | 3 | 4 | 5 |
| 19.I am able to adjust my emotions in a timely manner to maintain psychological stability. | 1 | 2 | 3 | 4 | 5 |
| 20.I will set clear goals to motivate myself to keep moving forward. | 1 | 2 | 3 | 4 | 5 |
| 21.I firmly believe that there is a positive side to everything. | 1 | 2 | 3 | 4 | 5 |
| 22.My emotional state often fluctuates violently and constantly changing. | 1 | 2 | 3 | 4 | 5 |
